# Activity and electron donor preference of two denitrifying bacterial strains identified by Raman gas spectroscopy

**DOI:** 10.1007/s00216-021-03541-y

**Published:** 2021-07-23

**Authors:** Annika Blohm, Swatantar Kumar, Andreas Knebl, Martina Herrmann, Kirsten Küsel, Jürgen Popp, Torsten Frosch

**Affiliations:** 1 Leibniz Institute of Photonic Technology, 07745 Jena, Germany; 2 Institute of Biodiversity, Friedrich Schiller University Jena, 07743 Jena, Germany; 3 German Centre for Integrative Biodiversity Research (iDiv) Halle-Jena-Leipzig, 04103 Leipzig, Germany; 4 Institute of Physical Chemistry, Friedrich Schiller University Jena, 07743 Jena, Germany; 5 Abbe Centre of Photonics, Friedrich Schiller University, 07743 Jena, Germany; 6 Biophotonics and Biomedical Engineering Group, Technical University Darmstadt, Merckstraße 25, 64283 Darmstadt, Germany

**Keywords:** Cavity-enhanced Raman spectroscopy, Denitrification, Multi-gas analysis, Nitrogen cycle

## Abstract

Human activities have greatly increased the input of reactive nitrogen species into the environment and disturbed the balance of the global N cycle. This imbalance may be offset by bacterial denitrification, an important process in maintaining the ecological balance of nitrogen. However, our understanding of the activity of mixotrophic denitrifying bacteria is not complete, as most research has focused on heterotrophic denitrification. The aim of this study was to investigate substrate preferences for two mixotrophic denitrifying bacterial strains, *Acidovorax delafieldii* and *Hydrogenophaga taeniospiralis*, under heterotrophic, autotrophic or mixotrophic conditions*.* This complex analysis was achieved by simultaneous identification and quantification of H_2_, O_2_, CO_2_, ^14^N_2_, ^15^N_2_ and ^15^N_2_O in course of the denitrification process with help of cavity-enhanced Raman spectroscopic (CERS) multi-gas analysis. To disentangle electron donor preferences for both bacterial strains, microcosm-based incubation experiments under varying substrate conditions were conducted. We found that *Acidovorax delafieldii* preferentially performed heterotrophic denitrification in the mixotrophic sub-experiments, while *Hydrogenophaga taeniospiralis* preferred autotrophic denitrification in the mixotrophic incubation. These observations were supported by stoichiometric calculations. The results demonstrate the prowess of advanced Raman multi-gas analysis to study substrate use and electron donor preferences in denitrification, based on the comprehensive quantification of complex microbial gas exchange processes.

**Supplementary Information:**

The online version contains supplementary material available at 10.1007/s00216-021-03541-y.

## Introduction

The global nitrogen cycle is a central biogeochemical cycle and crucial for life on earth. The significance of nitrogen to the biosphere and cellular life is indisputable; however, our fundamental knowledge of the microorganisms and enzymatic processes that transform nitrogen into its various oxidation states is still evolving. Different microbial processes are responsible for converting and balancing atmospheric dinitrogen (N_2_) and reactive and thus bioavailable nitrogen species in the biogeosphere [1–6]. Recent human activities unbalanced the nitrogen cycle as the burning of fossil fuels and the application of fertilizers heavily increased the input of reactive nitrogen to terrestrial and aquatic ecosystems [1, 2]. The resulting imbalance and accumulation of reactive nitrogen are harmful for both nature and humans. It can cause eutrophication, acidification and a loss in biodiversity and can lead to severe diseases in humans (e.g. methemoglobinemia) [1–4]. Denitrification is an important process in maintaining the ecological balance of nitrogen, as it converts reactive nitrogen to N_2_ gas, removing reactive nitrogen from hydrosphere and geosphere and releasing N_2_ into the atmosphere. Although denitrification was thought to be exclusively performed by prokaryotes (bacteria and archaea), eukaryotic organisms such as fungi and foraminifera also mediate this process and collectively play an important role in the global biogeochemical nitrogen cycle [5, 7–9]. In denitrifying bacteria, the reactive species nitrate (NO_3_^−^) and nitrite (NO_2_^−^) are transformed to N_2_ via several enzymatic reactions. A key to understand the current status of the nitrogen cycle is to what extent denitrification is influenced and can keep up with increasing availability of its substrate nitrate [1, 2]. Over the last decades, much attention has been paid to assess denitrification rates in a broad range of habitats [1–4], and denitrifying bacteria have been isolated from several ecosystems and were further investigated under laboratory conditions [5]. However, the effect of the type and availability of electron donors on the denitrifying process and denitrifying communities is still poorly understood [10, 11].

Denitrification is a four-step process transforming nitrate into N_2_ in sub-oxic and anoxic environments. The process is catalysed by several enzymes responsible for the different steps [5, 6].

1 $${{\mathrm{N}\mathrm{O}}_3}^{-} \rightarrow {{\mathrm{N}\mathrm{O}}_2}^{-}\rightarrow \mathrm{NO} \rightarrow {\mathrm{N}}_2\mathrm{O} \rightarrow {\mathrm{N}}_2$$

If denitrification is coupled to a heterotrophic lifestyle (later referred to as “heterotrophic denitrification”), an organic carbon compound is needed as electron donor and carbon source. A general equation showing substrate conversion and production in heterotrophic denitrification is as follows [12]:

2 $${\mathrm{C}}_{\mathrm{a}}{\mathrm{H}}_{\mathrm{b}}{\mathrm{O}}_{\mathrm{c}}+x\ {\mathrm{H}\mathrm{NO}}_2\rightarrow a\ {\mathrm{C}\mathrm{O}}_2+y\ {\mathrm{N}}_2+z\ {\mathrm{H}}_2\mathrm{O}$$

With *x* = 4/3 *a* + 1/3 *b* − 2/3 *c*

y = 2/3 *a* + 1/6 *b* − 1/3 *c*

*z* = 2/3 *a* + 2/3 *b* − 1/3 *c*

In contrast to this, denitrification coupled to a chemolithoautotrophic lifestyle (later referred to as “autotrophic denitrification”) uses carbon dioxide or bicarbonate as sole carbon source, as the organisms can convert it into organic carbon compounds themselves via CO_2_ fixation. Hydrogen, reduced iron or sulphur species are common electron donors in autotrophic denitrification [13–16]. Autotrophic denitrifying bacteria can prevail in organic carbon-limited ecosystems, utilizing inorganic electron donors such as hydrogen [17–19]. Oligotrophic groundwater is one type of habitat in which usually only a small amount of organic carbon is available. Under these conditions, chemolithoautotrophic lifestyles linked to denitrification are likely to become more competitive [20]. These autotrophic processes themselves and the biomass built up by chemolithoautotrophic primary production can also serve as a source of organic carbon for other biota and potentially support more complex food webs [21, 22].

Using hydrogen as electron donor, reactions taking place in denitrification are as follows [13]:

3 $$2\ {{\mathrm{NO}}_3}^{-}+2\ {\mathrm{H}}_2 \rightarrow 2\ {{\mathrm{NO}}_2}^{-}+2\ {\mathrm{H}}_2\mathrm{O}$$

4 $$2\ {{\mathrm{NO}}_2}^{-}+2\ {\mathrm{H}}^{+}+{\mathrm{H}}_2 \rightarrow 2\ \mathrm{NO}+2\ {\mathrm{H}}_2\mathrm{O}$$

5 $$2\ \mathrm{NO}+{\mathrm{H}}_2 \rightarrow {\mathrm{N}}_2\mathrm{O}+{\mathrm{H}}_2\mathrm{O}$$

6 $${\mathrm{N}}_2\mathrm{O}+{\mathrm{H}}_2\rightarrow {\mathrm{N}}_2+{\mathrm{H}}_2\mathrm{O}$$

∆*G*°: −240 kJ mol^−1^ [23]

Thus, five molecules of hydrogen are needed to completely reduce two molecules of nitrate to one molecule of nitrogen. Along with that, protons are consumed.

Microbes that can thrive on organic as well as inorganic carbon as carbon source are called mixotrophs, which also applies to a large number of denitrifying taxa. Depending on substrate availability, denitrification by these organisms can be linked to either an autotrophic or a heterotrophic lifestyle. In a truly mixotrophic lifestyle, the denitrifying bacteria use the inorganic electron donor for energy generation but use the organic compound as carbon source [24–26].

For a detailed analysis of denitrification processes and preferential substrate utilization, monitoring concentration changes of gases such as N_2_, N_2_O, CO_2_ and O_2_ is a prerequisite. Moreover, if hydrogen is used as electron donor in autotrophic denitrification, its consumption also needs to be tracked. Usually, isotopically labelled reactive nitrogen sources (typically ^15^N-nitrate) are used to unambiguously determine the amount of N_2_ and intermediate products like N_2_O produced during denitrification. In addition, labelled carbon sources can be used to determine the fate of the carbon utilized by the bacteria. Thus, the concentration changes of ^(15)^N_2_, ^(15)^N_2_O, ^(13)^CO_2_, H_2_ and O_2_ have to be observed for a comprehensive investigation of the denitrification process and substrate uses. Consequently, monitoring the consumption of electron donors and electron acceptors and generation of products and by-products requires a quantitative analysis of headspace gases with sufficient time resolution. This analysis should cover a wide concentration range with high sensitivity and selectivity, allowing, e.g., the discrimination between ^15^N_2_ produced from ^15^NO_3_^−^ in the denitrification process and ^14^N_2_ present in the measuring setup.

In order to follow changes in the suite of gases (N_2_, N_2_O, CO_2_ and O_2_), a selective multi-gas analysis is needed. Raman spectroscopy is an emerging analytical technique [27–29]. Due to the low sensitivity of conventional Raman scattering, elaborated enhancement techniques based on optical cavities [30, 31] and hollow fibres [32–34] were recently developed and applied in several environmental gas experiments [35, 36]. Various gases can be detected in parallel due to the substance-specific spectral position of the Raman bands and no gases are consumed, enabling continuous in situ measurements [37–39]. Thus, Raman spectroscopy is ideally suited to investigate metabolic processes, such as denitrification. Concentration changes of complex gas mixtures including isotopically labelled gases can be monitored.

In this study, we used cavity-enhanced Raman spectroscopy (CERS) to investigate substrate use and substrate preference of two mixotrophic denitrifying hydrogenotrophic bacterial strains *Acidovorax delafieldii* strain 16 and *Hydrogenophaga taeniospiralis* strain 2K1, which are representative of denitrifiers in oligotrophic groundwater at the CZE (Critical Zone Exploratory) Hainich, Germany [40, 41]. Our goals were (i) to compare denitrification activity and growth of the two strains under heterotrophic, autotrophic and mixotrophic growth conditions; (ii) to identify which electron donor was preferentially used under mixotrophic conditions; and (iii) to evaluate the suitability of CERS to get a comprehensive insight into the microbial transformation processes in these incubations.

## Materials and methods

### Bacterial strains, media and incubation conditions

Two bacterial strains were used in incubation experiments under denitrifying conditions with either hydrogen or organic electron donors. *Acidovorax delafieldii* strain 16 was isolated from oligotrophic oxic groundwater from the Hainich CZE using a traditional gelrite-shake dilution approach. In brief, groundwater (50 μL) was inoculated into Hungate tubes containing 20 mL of nitrate-thiosulfate-carbonate (NTC) mineral medium [40] with 0.5% Gelrite under a gas atmosphere of 80% N_2_, 10% CO_2_ and 10% H_2_ and incubated under dark conditions at 15 °C. After 4 weeks, bacterial colonies developed in the semisolid medium were picked with sterile Pasteur pipettes and inoculated into Gelrite-free NTC mineral media. Incubations were carried out at 15 °C in the dark for a period of 1 month followed by a repeated process for 26 consecutive transfers with alternate semisolid and liquid NTC mineral medium. *Hydrogenophaga taeniospiralis* strain 2K1 DSM 2082 was obtained from the Deutsche Sammlung von Mikroorganismen und Zellkulturen (DSMZ). All incubations were carried out in 120 mL serum bottles with 60-mL medium, sealed with butyl rubber stoppers and aluminum crimps.

For the microcosm experiment, *A. delafieldii* strain 16 was grown at 15 °C in modified ATCC medium 1246 with the following composition: 1.5 g L^−1^ KH_2_PO_4_, 1.5 g L^−1^ Na_2_HPO_4_ * 2 H_2_O, 0.1 g L^−1^ NH_4_Cl, 0.5 g L^−1^ MgSO_4_ * 7 H_2_O, 0.1 g L^−1^ CaCl_2_ * 2 H_2_O, 0.5 g L^−1^ MgCl_2_ * 6 H_2_O, 0.01 g L^−1^ MnCl_2_, 0.01 g L^−1^ FeCl_3_, 10 μM CuCl_2_, 10 mM NaHCO_3_, vitamin solution [DSMZ 461 Mineral medium (Nagel and Andreesen): http://www.dsmz.de] (5 mL L^−1^) and trace element solution [DSMZ 461 Mineral medium (Nagel and Andreesen): http://www.dsmz.de] (1 mL L^−1^) per 1-L medium.

*Hydrogenophaga taeniospiralis* strain 2K1 was grown at 27 °C in DSMZ medium 81 (mineral medium for chemolithoautotrophic growth).

For the setup of each microcosm experiment, a preculture of each strain was set up in triplicate 120 mL serum vials. Cells were grown for 1 week at 15 °C (*A. delafieldii* strain 16) or 3 days at 27 °C (*H. taeniospiralis* strain 2K1) using the mineral media described above with 3 mmol L^−1^ (*A. delafieldii* strain 16) or 2 mmol L^−1^ NaNO_3_ (98% ^15^N) (*H. taeniospiralis* strain 2K1) and 3 mmol L^−1^ acetate or 1 mmol L^−1^ mannitol, respectively. Precultures were harvested by centrifugation in sterile 50 mL centrifuge tubes at 4000 * g and 15 °C for 6 min and washed twice with the respective mineral medium (without nitrate or carbon source) used for each strain. Subsequently, cell pellets were resuspended in mineral medium and were used to inoculate the denitrification microcosm experiments to an initial OD (600 nm) of 0.002.

For *A. delafieldii* strain 16, NaNO_3_ (98% ^15^N) was added to a final concentration of 3 mmol L^−1^. For heterotrophic and mixotrophic incubation conditions, sodium acetate (^13^C_2_H_3_NaO_2_) was added to a final concentration of 3 mmol L^−1^. The serum bottles for heterotrophic growth conditions were flushed with a gas mixture of 10% CO_2_ and 90% Ar (argon) to establish a defined headspace. For incubations under mixotrophic and autotrophic conditions, the headspace was composed of 10% CO_2_, 20% H_2_ and 70% argon. The sample bottles were incubated at 15 °C in the dark.

To the sample bottles of *H. taeniospiralis* strain 2K1, NaNO_3_ (98% ^15^N) was added to a final concentration of 2 mmol L^−1^. For growth under heterotrophic and mixotrophic conditions, mannitol was added to a final concentration of 1 mmol L^−1^. For growth under mixotrophic conditions, nitrate was replenished to a final concentration of 2 mmol L^−1^ after 160 and 209 h of incubation. Incubations were flushed with 10% CO_2_ and 90% argon for heterotrophic incubation conditions and with 10% CO_2_, 50% H_2_ and 40% argon for mixotrophic and autotrophic incubation conditions to establish the headspace. Sample bottles were incubated at 30 °C in the dark.

### Raman spectroscopic gas analysis

Headspace gas analysis was performed using cavity-enhanced Raman spectroscopy (CERS). As described previously [42], an optical power build-up cavity with highly reflective mirrors was used to enhance the intensity of the laser diode (*λ* = 650 nm, *P*_laser_ = 50 mW) by several orders of magnitude up to 100 W. This enhancement of the laser power enabled the measurement of the gas concentrations between about 100 ppm and 100%. The sample gas was passed through this cavity and both pressure and temperature were recorded via sensors integrated into the spectrometer. Gas pre-treatment was not necessary, and no gases were consumed during the measurement. Thus, continuous measurements in a closed cycle were possible.

A robust calibration of the spectrometer could be realized due to the linear relationship between Raman scattering intensity and concentration. A spectrum of pure Raman-inactive argon was first acquired, which was used for background correction in subsequent measurements. Afterwards, spectra of the pure gases, which were expected in the experiment (H_2_, CO_2_, ^13^CO_2_, ^15^N_2_O, ^15^N_2_, O_2_, N_2_), were taken and further used as reference spectra. For concentration calculation, a multilinear regression using the full set of reference spectra and the measured spectrum was applied. This evaluation method basically expresses the measured spectrum as the sum of the weighted reference spectra, which results in weighting factors of the single gases. These weighting factors are proportional to the volumetric mixing ratio of the single gases.

### Gas analysis setup

**Fig. 1:**
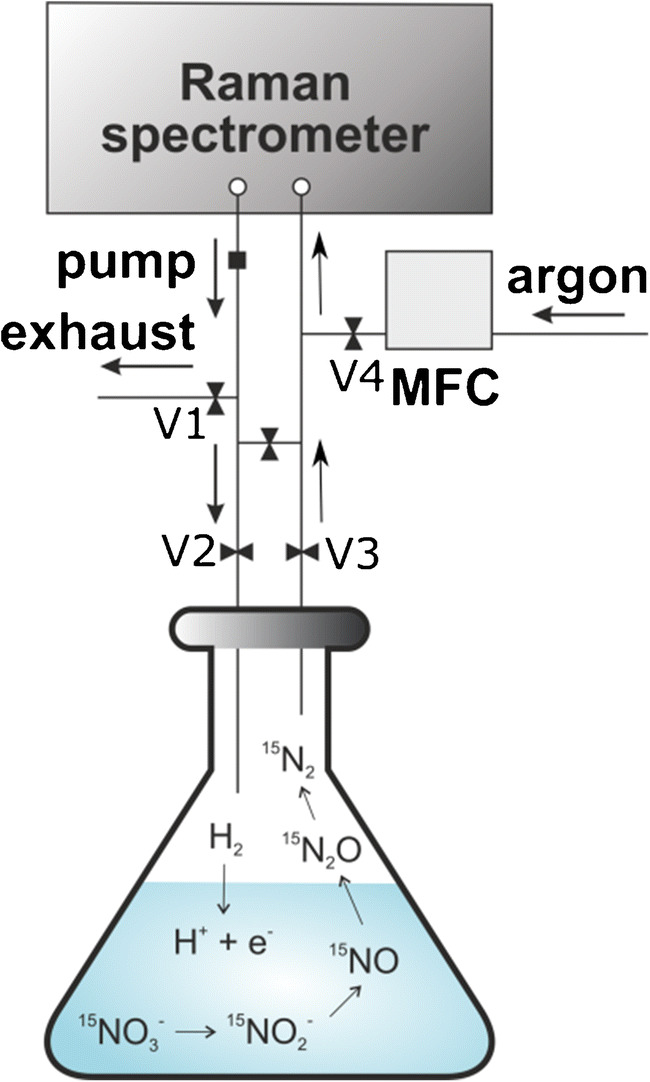
Gas analysis setup for the denitrification experiments. A closed cycle between the sample and the Raman spectrometer was established for measurements, in which the sample headspace could be cycled (V1 and V4 closed, V2 and V3 open). Argon was connected to flush the measurement cycle in between measurements using MFCs (mass flow controllers); in this case, V1 and V4 are open and V2 and V3 are closed

The measurement setup for the headspace analysis is shown in Fig. 1. Before and in between measurements, the setup was flushed with the Raman-inactive gas argon until no Raman peaks were visible anymore. Then, the sample was connected to the setup and a closed measurement cycle was established. The gases were measured for 5 min while being constantly circulated. Complete mixing of argon in the measurement setup and the headspace was realized in less than 1 min. As the exact volume of the measurement setup (40 mL) and the headspace (60 mL) were known as well as the temperature and the pressure in the system, the original concentration of analysed gases in the bottle headspace could be calculated.

As hydrogen can diffuse very easily, a correction for diffusion was used here. For each measurement time point, the difference between the control and replicate samples was calculated. This was done under the assumption that all samples of one time point have lost a comparable amount of hydrogen due to diffusion. The control samples lose hydrogen only due to diffusion, while the replicate samples lose hydrogen due to diffusion and denitrification. Thus, the correct amount of hydrogen used in denitrification is the difference between control and replicate samples.

### Liquid medium analysis

After measurement of headspace gases, *Acidovorax delafieldii* strain 16 cultures were centrifuged at 20,000 * g and 4 °C for 20 min. The supernatant was transferred to a new tube and frozen at −20 °C for later analysis of nitrate and nitrite. Optical density measurements could not be performed for this bacterial strain due to the flocculation type growth behaviour in the liquid medium.

Immediately after the headspace measurement of *Hydrogenophaga taeniospiralis* strain 2K1, 1.5 mL of the medium was removed for OD measurement. The samples were measured (Jasco UV/VIS/NIR, V-670, wavelength 600 nm) using pure medium as a reference. Additionally, 10 mL medium was transferred into 15 mL Falcon tubes, centrifuged at 5000 rpm (Herolab UniCen MR) for 15 min. The supernatant was transferred to a new 15 mL Falcon tube and was frozen at −20 °C until further analysis for nitrate, nitrite and mannitol.

Concentrations of nitrate and nitrite in the incubations were determined by colorimetric assays using the salicylate method [43] and the Griess method [44], respectively. For the *Hydrogenophaga taeniospiralis* strain K21 incubations, no reliable determination of nitrate could be achieved in presence of hydrogen using the respective method.

Mannitol analysis was performed on GC-MS, for a detailed description, see Supplementary Information (ESM) - Methods. A strong matrix effect was observed resulting in much lower than expected values. As for nitrate, no reliable mannitol concentrations could be obtained in the presence of H_2_.

## Results and discussion

Cavity-enhanced Raman spectroscopy (CERS) is ideally suited for the monitoring of the denitrification process. This is demonstrated by an exemplary spectrum, where all components of the sample headspace (except Raman-inactive argon) are spectrally well resolved and can be analysed simultaneously (see ESM Fig. S1).

The two characteristic peaks of the Fermi dyad of CO_2_ can be seen at ${\overset{\sim }{\nu}}_{-}=1285\ {\mathrm{cm}}^{-1}$ and ${\overset{\sim }{\nu}}_{+}=1388\ {\mathrm{cm}}^{-1}$ [45]. For ^13^CO_2_, these peaks are at ${\overset{\sim }{\nu}}_{-}=1265\ {\mathrm{cm}}^{-1}$ and ${\overset{\sim }{\nu}}_{+}=1370\ {\mathrm{cm}}^{-1}$ overlap with ^12^CO_2_ in the spectrum if both gases are present in the headspace at the same time. Using linear combinations of calibration spectra of ^12^CO_2_ and ^13^CO_2_, the concentrations of both isotopologues can be gained from the Raman spectra [46, 47]. H_2_ has rotational bands at $\overset{\sim }{\nu }\ \left({S}_0(1)\right)=587\ {\mathrm{cm}}^{-1}$, $\overset{\sim }{\nu }\ \Big({S}_0(2)=814\ {\mathrm{cm}}^{-1}$ and $\overset{\sim }{\nu }\ \Big({S}_0(3)=1034\ {\mathrm{cm}}^{-1}$ [48]. ^15^N_2_ at ${\overset{\sim }{\nu}}_0=2252\ {\mathrm{cm}}^{-1}$ [36] can be clearly discriminated from ^14^N_2_ (${\overset{\sim }{\nu}}_0=2331\ {\mathrm{cm}}^{-1}$) [45], which enters the measurement setup together with O_2_ (${\overset{\sim }{\nu}}_0=1556\ {\mathrm{cm}}^{-1}$) [45] via diffusion in small amounts. No additional compounds like the intermediate product ^15^N_2_O ($\overset{\sim }{\nu }=1265\ {\mathrm{cm}}^{-1}$ and $\overset{\sim }{\nu }=2155\ {\mathrm{cm}}^{-1}$) [36] could be seen in the spectra (for the example spectrum, see ESM Fig. S1).

### Denitrification and electron donor preference of *Acidovorax delafieldii* strain 16

*Acidovorax delafieldii* strain 16 was isolated from groundwater samples from the Hainich CZE. Other strains of this bacterial species have been described as mixotrophic bacteria with differing properties, especially regarding denitrification [49]. To assess the metabolic capabilities of the strain from the Hainich CZE, *Acidovorax delafieldii* strain 16 was cultured under three different growth conditions to investigate the growth dynamics under heterotrophic, autotrophic and mixotrophic conditions. In the experiments under heterotrophic and mixotrophic growth conditions, concentrations of ^15^N_2_ and ^13^CO_2_ in the headspace increased with time, indicating complete denitrification to N_2_ by oxidation of the organic electron donor. Under autotrophic growth conditions, no bacterial growth or substrate conversion was observed. Since the initial isolation and subsequent testing for denitrification activity, the strain had apparently lost its ability to use hydrogen as electron donor in denitrification.

**Fig. 2:**
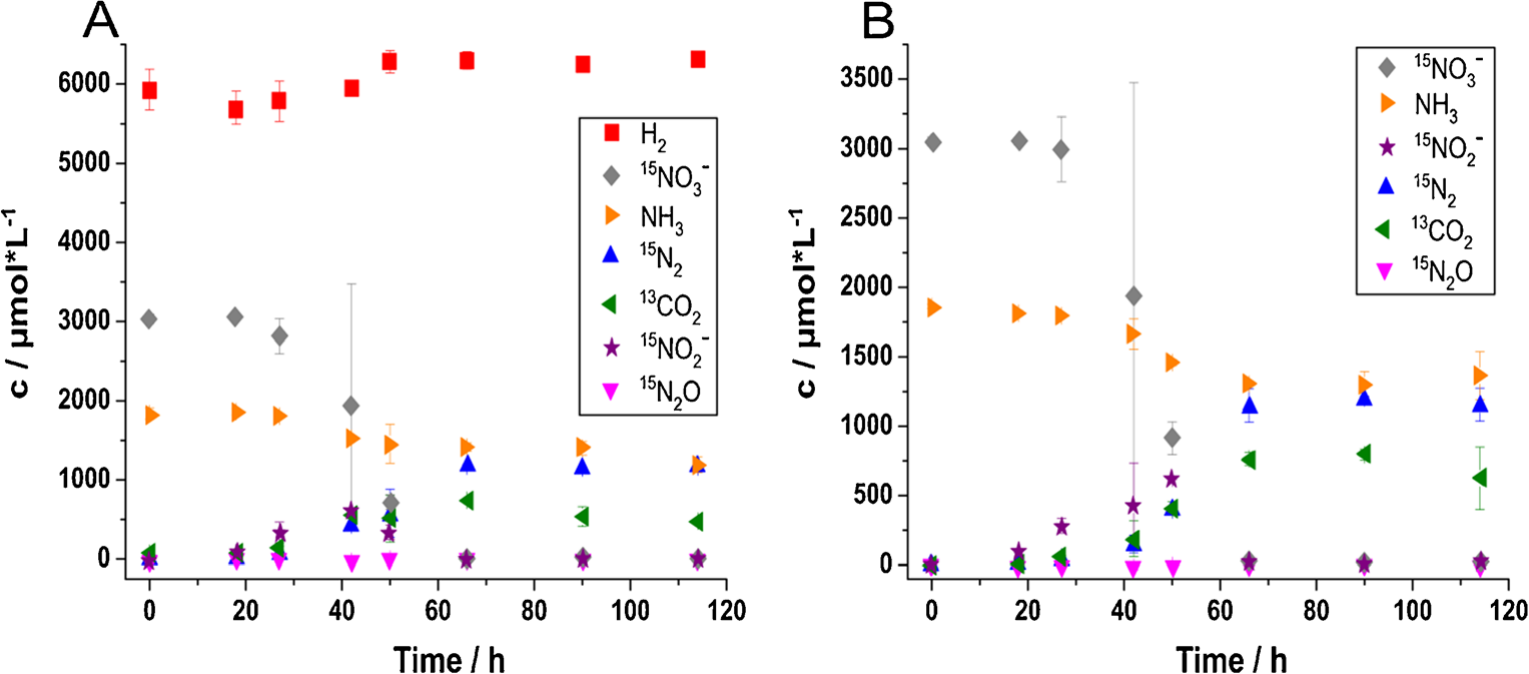
Concentration changes of selected gases in the headspace and of ^15^NO_3_^−^, ^15^NO_2_^−^ and ammonium (NH_4_^+^) in the liquid phase of *Acidovorax delafieldii* strain 16 cultures under different incubation conditions over time. **A** Mixotrophic conditions, both H_2_/CO_2_ and ^13^C-acetate are available as substrates. **B** Heterotrophic conditions, ^13^C-acetate is available as substrate. Depicted values are averaged measurements from three replicate culture flasks with standard deviation

The use of nitrate and hydrogen as well as the production of gaseous end products could be followed closely for the experiments under heterotrophic and mixotrophic conditions (Fig. 2). Nitrate was depleted in the liquid phase after 65 h, when all the nitrite, which had built up after around 50 h of incubation, had been consumed, too (Fig. 2A and B). At that time point, the maximum ^15^N_2_ headspace concentration (about 1200 μmol L^−1^) was detected (Fig. 2A and B). After that, no further increase was seen, suggesting complete conversion of nitrate to dinitrogen gas. The large standard deviation for nitrate at 40 h in Fig. 2A and B can be explained by slightly different growth dynamics of the bacteria in the three individual replicate incubation flasks which were sampled at this time point. During the exponential phase, nitrate concentrations decreased rapidly within just 20 h. Consequently, minor differences in the progress of bacterial activity within each flask resulted in large differences in nitrate concentrations between the replicates. The concentration change in nitrate and dinitrogen gas was mirrored by ^13^CO_2_ concentration changes in the gas phase, where about 700 μmol L^−1^ was produced (Fig. 2A and B) from the utilization of ^13^C-labelled acetate as organic electron donor. Hydrogen gas was available as alternative electron donor in the incubations under mixotrophic conditions; however, only minor concentration changes of hydrogen were observed (Fig. 2A).

**Fig. 3:**
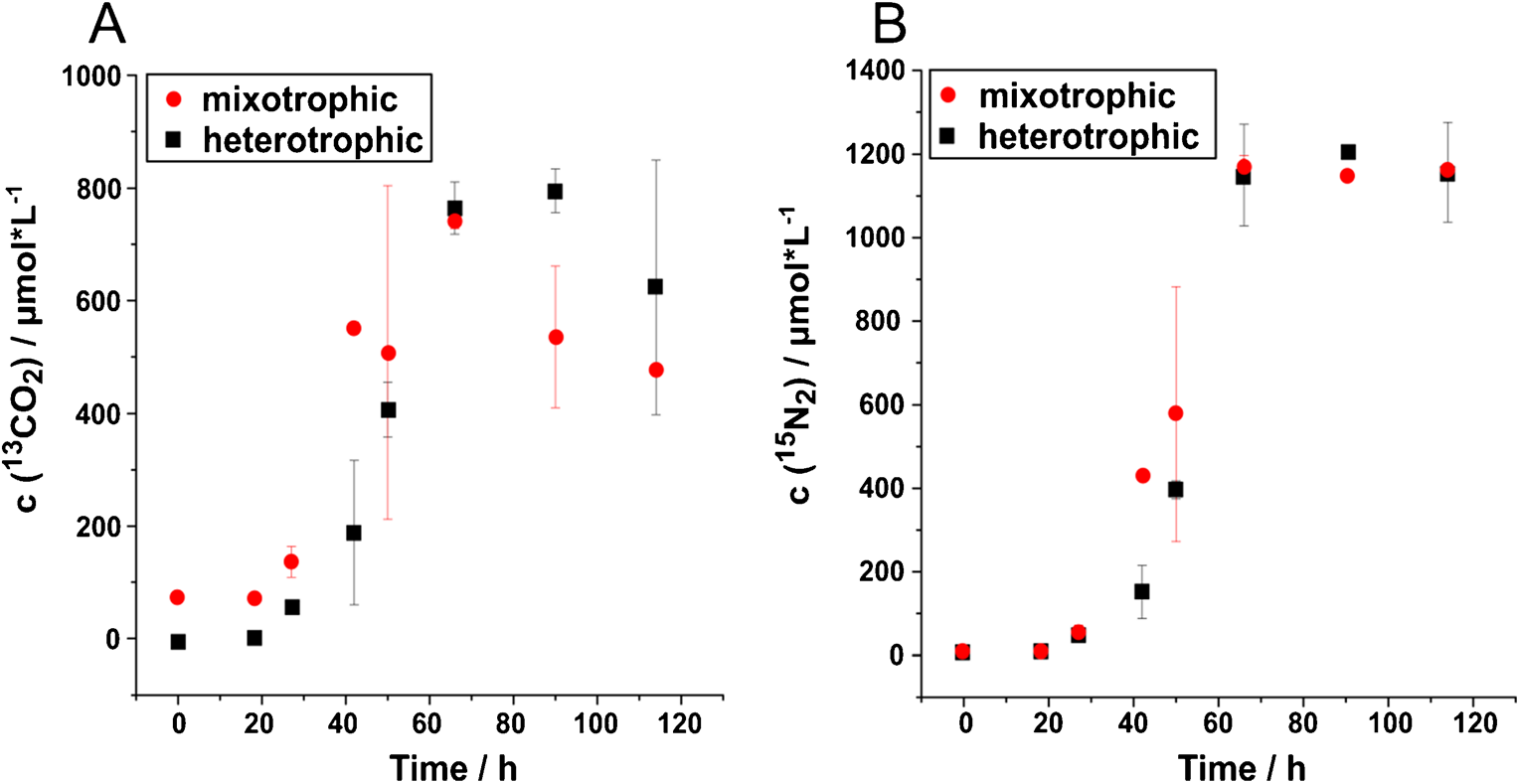
Comparison of concentration changes over time for **A** ^13^CO_2_ and **B** ^15^N_2_ under heterotrophic and mixotrophic growth conditions for *Acidovorax delafieldii* strain 16. For both gases, concentration changes and time course are very similar for the two incubations. Depicted values are averaged measurements from three replicate culture flasks with standard deviation

Concentration changes for ^13^CO_2_ (Fig. 3A) and ^15^N_2_ (Fig. 3B) were compared for heterotrophic and mixotrophic growth conditions. Concentration changes as well as time course are very similar for both gases (Fig. 3). Similar final concentrations were reached for ^15^N_2_ (Fig. 3B) and ^13^CO_2_ (Fig. 3A) at the end of the incubations. Nitrogen production rates were calculated based on the near-linear concentration increase between 27 and 66 h (mixotrophic incubation) and 42 and 66 h (heterotrophic incubation). For the incubation under heterotrophic conditions, a denitrification rate of 42.38 μmol N_2_ L^−1^ h^−1^ was achieved, for the mixotrophic incubation only 28.27 μmol N_2_ L^−1^ h^1^ even though nitrogen production seems to have started a little earlier than for the heterotrophic incubation.

To determine whether heterotrophic or autotrophic denitrification prevailed under mixotrophic conditions, we performed stoichiometric calculations based on the added substrates and measured headspace gas concentrations. The adapted Eq. 1 for heterotrophic denitrification using acetate as organic substrate and sodium nitrate is as follows:

7 $$8\ {\mathrm{N}\mathrm{a}}^{15}{\mathrm{N}\mathrm{O}}_3+{5}^{13}{\mathrm{C}}_2{\mathrm{H}}_4{\mathrm{O}}_2\rightarrow {10}^{13}{\mathrm{C}\mathrm{O}}_2+{4}^{15}{\mathrm{N}}_2+6\ {\mathrm{H}}_2\mathrm{O}+8\ {\mathrm{O}\mathrm{H}}^{-}+8\ {\mathrm{N}\mathrm{a}}^{+}$$

A final ^15^N_2_ concentration of 1200 μmol L^−1^ indicated that out of the original 3 mmol nitrate L^−1^, 2.4 mmol L^−1^ had been converted to N_2_. The missing fraction of 0.6 mmol L^−1^ might have been consumed as nitrogen source for biomass build-up by assimilatory nitrate reduction. However, this explanation is unlikely, due to the fact that the medium also contained ammonium which serves as nitrogen source and was not depleted in the course of the incubations. Consequently, the reason remains unclear why not the entire nitrate was retrieved as N_2_ in the final gas phase. Liquid-phase measurements showed that no nitrate or nitrite remained in the medium, providing further support for a complete denitrification process under both heterotrophic and mixotrophic conditions.

The reduction of 2.4 mmol L^−1^ nitrate by denitrification would have required the consumption of 1.5 mmol L^−1^ of the added 3 mmol L^−1^ of ^13^C-actetate and the production of 3 mmol L^−1 13^CO_2_. In the gas phase, we could measure an increase of 0.7 mmol L^−1 13^CO_2_, which means that 2.3 mmol L^−1^ remained in the liquid phase. This can be explained with the carbonate buffer system (see Eq. 8) active in the incubations. In denitrification, protons are consumed, and alkalinity increases in the reduction step of nitrite to NO (see Eq. 4), which in turn leads to carbon dioxide being dissolved and dissociated to keep the buffer in equilibrium.

8 $${\mathrm{CO}}_2+{\mathrm{H}}_2\mathrm{O}\rightarrow {\mathrm{H}}_2{\mathrm{CO}}_3\rightarrow {{\mathrm{H}\mathrm{CO}}_3}^{-}+{\mathrm{H}}^{+}$$

### Denitrification and electron donor preference of *Hydrogenophaga taeniospiralis* strain 2K1

*Hydrogenophaga taeniospiralis* strain 2K1 is a mixotrophic bacterium which is known to only use hydrogen as electron donor in autotrophic metabolism [50]. To investigate whether *Hydrogenophaga taeniospiralis* strain 2K1 prefers to utilize organic or inorganic electron donors when grown under mixotrophic conditions, different incubation experiments were established. Mannitol served as electron donor and carbon source for heterotrophic growth under heterotrophic and mixotrophic conditions, while hydrogen acted as electron donor for autotrophic and mixotrophic growth under autotrophic and mixotrophic conditions.

**Fig. 4:**
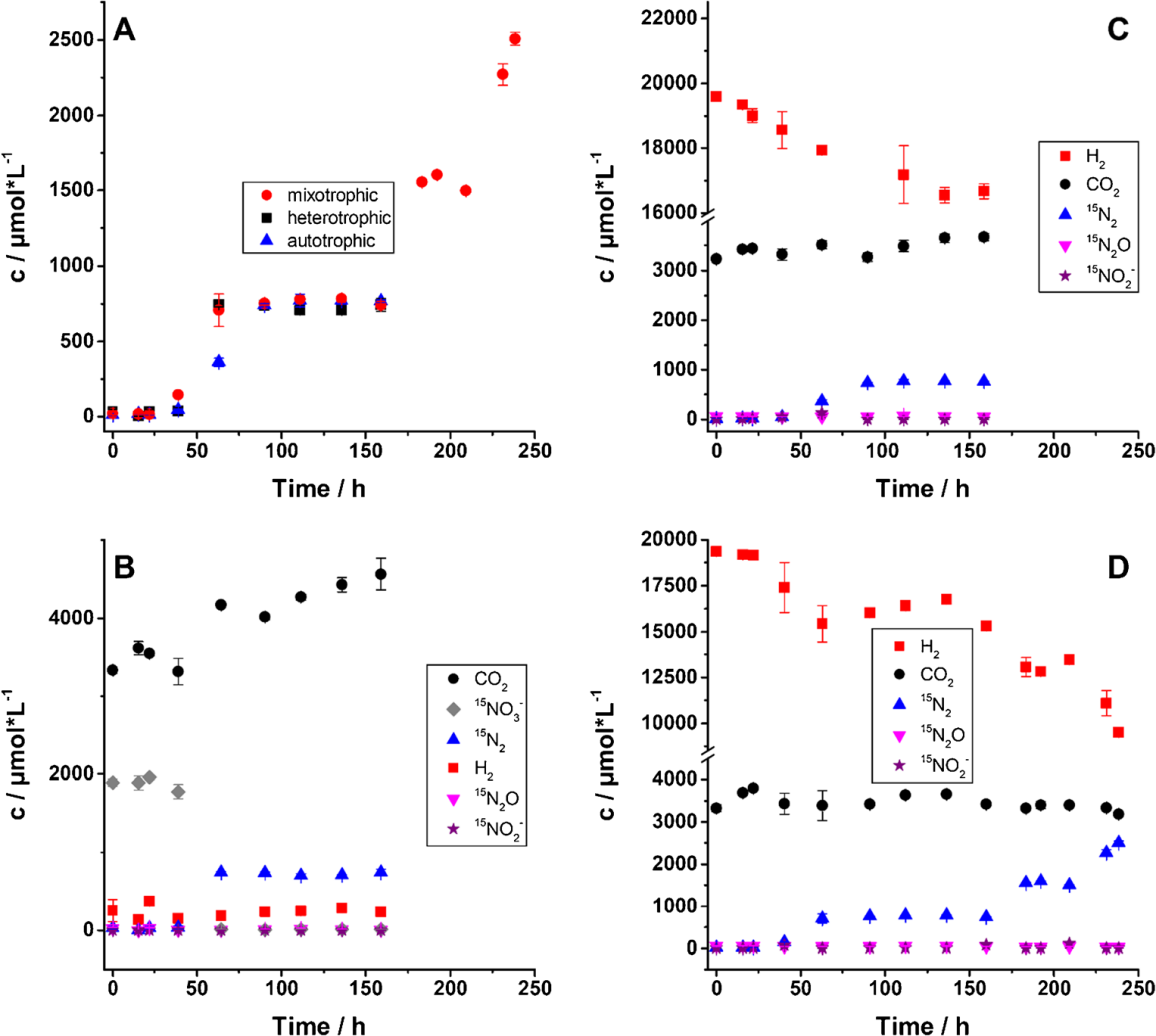
Concentration changes of headspace gases and NO_3_^−^ and NO_2_^−^ in the liquid medium over time for denitrification by *Hydrogenophaga taeniospiralis* strain 2K1 under three different growth conditions. **A** ^15^N_2_ concentrations. **B** All gases as well as nitrate and nitrite, heterotrophic incubation. **C** All gases and nitrite, autotrophic conditions. **D** All gases and nitrite, mixotrophic conditions. Depicted values are averages with standard deviations

For *Hydrogenophaga taeniospiralis* strain 2K1, complete denitrification was performed under all three different growth conditions (Fig. 4). The detected ^15^N_2_ headspace concentrations were in good agreement with the expected values based on the amount of ^15^N-nitrate added to the medium. For growth and denitrification under autotrophic conditions, ^15^N_2_ concentrations were lower than for the other two experimental setups during the exponential phase after 63 h incubation (Fig. 4A). ^15^N_2_O was not detected in the headspace and no significant amounts of nitrite (maximum of 142 μmol L^−1^ at 63 h in the autotrophic incubation) were detected in the medium in any incubation (Fig. 4B–D). For the experiment under heterotrophic conditions, ^15^N_2_ production coincided with an increase in CO_2_ concentrations and nitrate was depleted after 63 h, when ^15^N_2_ concentrations had increased strongly (Fig. 4B).

Even though measured mannitol concentrations were much lower than expected due to a strong matrix effect, the general behaviour can be deduced. Mannitol concentrations decreased over the course of the experiment with the decrease being the largest, when N_2_ production rates were the highest. Mannitol was not completely depleted but reached a plateau at about 0.5 μmol L^−1^ (see ESM Fig. S2). A small increase in H_2_ concentrations was also visible (Fig. 4B). In the experiment under autotrophic conditions, ^15^N_2_ production was paralleled by a decrease in H_2_ concentrations (Fig. 4C). CO_2_ concentrations did not change over the course of the measurements (Fig. 4C). Under mixotrophic growth conditions, ^15^N_2_ was produced while H_2_ concentrations decreased, and CO_2_ remained at a stable level (Fig. 4D).

Nitrogen production rates were calculated for *Hydrogenophaga taeniospiralis* strain 2K1 based on the near-linear concentration changes during the exponential phase. For the heterotrophic incubation, a rate of 27.95 μmol N_2_ L^−1^ h^−1^ was achieved (between 39 and 63 h) and for the autotrophic incubation 13.69 μmol N_2_ L^−1^ h^−1^ (between 39 and 90 h). Due to the additional doses of nitrate, three rates could be calculated for the mixotrophic incubation. These rates increased over time in the experiment and are the highest found in this experiment. 24.75 μmol N_2_ L^−1^ h^−1^ (between 22 and 63 h), 34.67 μmol N_2_ L^−1^ h^−1^ (between 158 and 183 h) and 34.69 μmol N_2_ L^−1^ h^−1^ (between 209 and 238.5 h) were calculated. These nitrogen production rates are similar to those of *Acidovorax delafieldii* (42.38 μmol N_2_ L^−1^ h^−1^ for the heterotrophic incubation and 28.27 μmol N_2_ L^−1^ h^−1^ for the mixotrophic incubation) and are in the same range as those reported in Kumar et al. 2018 (243 μmol L^−1^ day^−1^, which corresponds to 10.125 μmol L^−1^ h^−1^, [40]).

**Fig. 5:**
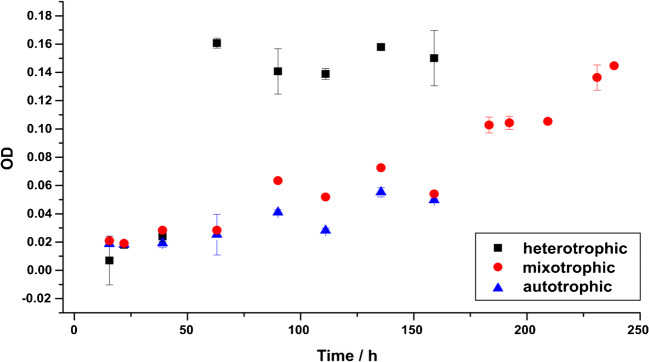
Optical density (OD) for incubations with *Hydrogenophaga taeniospiralis* strain 2K1 under three different growth conditions. Depicted values are averages from measurements of three replicate culture flasks with standard deviation

For each measured sample, the optical density (OD) was determined (Fig. 5). Under heterotrophic conditions, OD values increased more rapidly over time, suggesting that biomass built up much faster than in the other two incubations (Fig. 5), which is in accordance with other experiments reported in literature [13, 51, 52]. While incubations under heterotrophic and autotrophic conditions were stopped after 180 h when nitrate was depleted, additional nitrate was added to the incubations under mixotrophic conditions, allowing denitrification to continue and OD values and thus bacterial biomass still to increase (Figs. 4A and 5).

To gain more insight which electron donor or carbon source was preferentially used under mixotrophic growth conditions, stoichiometric calculations were conducted for the *Hydrogenophaga taeniospiralis* experiments. The calculations are based on the added substrates and the measured headspace concentrations. When mannitol is used as organic substrate in heterotrophic denitrification, the equation shown in “Introduction” (Eq. 1) is as follows [12]:

10 $$5\ {\mathrm{C}}_6{\mathrm{H}}_{14}{\mathrm{O}}_6+26\ {\mathrm{H}\mathrm{NO}}_3\rightarrow 30\ {\mathrm{C}\mathrm{O}}_2+13\ {\mathrm{N}}_2+48\ {\mathrm{H}}_2\mathrm{O}$$

A ^15^N_2_ concentration of about 0.75 mmol L^−1^ was measured in the gas phase, which accounts for ¾ of the added nitrate in the medium (1.5 mmol L^−1^ of 2 mmol L^−1^). Similar to the experiment with *Acidovorax delafieldii* strain 16, it remains unclear whether part of this nitrate was used as nitrogen source for biomass build-up. For heterotrophic denitrification, an increase in ^15^N_2_ concentrations by 0.75 mmol L^−1^ would be accompanied by an increase in CO_2_ concentrations in the headspace by 1.730 mmol L^−1^ and a decrease in mannitol concentrations in the medium by 0.290 mmol L^−1^ from the original concentration of 1 mmol L^−1^. As explained for *Acidovorax delafieldii*, alkalinity increases in denitrification (Eq. 9), which leads to a decrease in CO_2_ concentrations to keep the carbonate buffer in equilibrium (Eq. 8). For the incubation under heterotrophic conditions, an increase of approximately 1.2 mmol L^−1^ in CO_2_ concentrations was observed. Thus, part of the CO_2_ produced during heterotrophic denitrification remained in the liquid phase. For incubations under mixotrophic and autotrophic sub-experiments, we did not observe an increase in CO_2_.

Mannitol was used in heterotrophic denitrification, but due to the high matrix effect influencing the GC-MS measurements, mannitol concentration values are not suited for rigorous calculations. However, the general behaviour can be deduced.

For hydrogenotrophic denitrification, 5 mmol of hydrogen is used up to produce 1 mmol of N_2_. For the observed increase in ^15^N_2_ concentrations by 0.750 mmol L^−1^, this would correspond to a reduction in the concentration of hydrogen by 3.750 mmol L^−1^. This fits very well with the measured hydrogen concentrations, where Δ c(H_2_) values were up to 3.787 mmol L^−1^ in the mixotrophic and autotrophic sub-experiments with an average value of 3.067 ± 0.632 mmol L^−1^ at the end of the experiment.

Taking these stoichiometric calculations and the measured data into account, it can be concluded that in the denitrification experiments under heterotrophic and autotrophic conditions, heterotrophic and autotrophic denitrification was performed, respectively. For the mixotrophic incubation, the case is not quite straightforward. A clear decrease in hydrogen concentrations in a similar range as for the autotrophic incubation was observed. For CO_2_, no concentration changes were observed for the mixotrophic and autotrophic incubations, but an obvious increase was measured for the heterotrophic incubation. Finally, OD values for the mixotrophic and autotrophic incubations were very similar with the mixotrophic values being slightly higher than the autotrophic ones. But for ^15^N_2_ concentrations, the mixotrophic data is more similar to the heterotrophic than to the autotrophic data, especially at 63 h. For the ^15^N_2_ production rates, the same observation was made.

Departing from these observations, it can be concluded that autotrophic denitrification took place in the mixotrophic incubations and hydrogen was preferred over mannitol as electron donor. The observed differences from the purely autotrophic incubations suggest a boost by mannitol as carbon source which cannot be confirmed by mannitol measurements for this setup.

To get a better overview of the amount of electron donors available to the bacteria, the concentration of hydrogen in the liquid medium was calculated using Henry’s law. The calculated hydrogen concentrations are by a factor of 1000 smaller than the maximum concentrations of mannitol, as the maximum dissolved hydrogen concentration was about 0.004 mmol L^−1^. This makes the preference of hydrogen over mannitol as electron donor even more remarkable, as literature usually speaks of facultative autotrophs [50], which is to say that they can perform autotrophic denitrification when the substrate for heterotrophic denitrification is not available. The heterotrophy is preferred over the autotrophy, because it is reported to be more energy efficient [53]. This contrasts with our results, where autotrophic denitrification was performed in the incubation under mixotrophic conditions.

### Comparison of substrate usage preferences

Stoichiometric calculations based on added substrates and produced gases showed that *Acidovorax delafieldii* strain 16 used the organic electron donor and carbon source in the sub-experiment under mixotrophic conditions. Thus, it can be concluded that *Acidovorax delafieldii* strain 16 performed heterotrophic denitrification in the mixotrophic incubation. This is as in accordance with literature, where heterotrophic denitrification is described as thermodynamically preferred over autotrophic denitrification [53]. As the trait for using hydrogen in denitrification was obviously lost in between preliminary experiments and the main experiment, no results were achieved for autotrophic denitrification.

For *Hydrogenophaga taeniospiralis* strain 2K1, fully heterotrophic denitrification was only carried out in the sub-experiment under heterotrophic growth conditions. In contrast, hydrogen was preferred as electron donor over mannitol in the sub-experiment under mixotrophic growth conditions. This means that the growth was supported by autotrophic denitrification. However, it is likely that mannitol has been used to a small extent in the mixotrophic incubation to enhance denitrification rates, even though H_2_ usage and growth measured via OD were more similar to the autotrophic incubations. Jewell et al. explained, that for mixotrophic growth, mainly heterotrophic denitrification takes place, but chemolithoautotrophy is used in combination with heterotrophy to gain advantage over purely heterotrophic growth [54].

In our experiments with *Hydrogenophaga taeniospiralis* strain 2K1, the autotrophic substrates are preferred over the heterotrophic ones, when offered at the same time. Autotrophic denitrification was performed, even though the concentration of the heterotrophic electron donor mannitol was 1000-fold higher than the concentration of dissolved hydrogen. Mannitol seems to have been used to boost denitrification rates compared to the purely autotrophic incubations.

Our lab experiments allow the clear determination of substrate preferences and growth characteristics of denitrifying bacteria. The transfer to natural systems is associated with uncertainties, as the complex environmental conditions cannot be mimicked in the laboratory. Furthermore, there is a high degree of diversity in bacteria performing denitrification in each ecosystem [5]. Every bacterial strain has optimum growth and performance conditions, which are adapted to the environment they were isolated from, but which can also change in prolonged incubation series. Sophisticated methods are needed to disentangle the complexity of metabolic processes in nature.

## Conclusion

CERS multi-gas analysis was applied for the comprehensive analysis of the headspace of two microbial cultures performing denitrification. Substrate consumption and gas production in denitrification were followed closely throughout the experiment. The gases H_2_, ^(13)^CO_2_, ^(15)^N_2_, ^15^N_2_O and O_2_ were simultaneously quantified. The ability for sensitive quantification of hydrogen and nitrogen is highlighted. The presence of unknown or unexpected gases could be excluded, as no additional peaks were detected in the Raman spectra. The denitrifying bacteria produced ^15^N_2_ from ^15^N-labelled nitrate. The stable isotope ^15^N was used to distinguish the nitrogen produced via denitrification from atmospheric nitrogen. The Raman peaks of ^14^N_2_ and ^15^N_2_ are well separated in the spectra; thus, both nitrogen species could be quantified, and diffusion effects could be separated from microbial processes (see ESM Fig. S1).

Substrate preference could be assigned for the separate experiments and heterotrophic and autotrophic denitrification could be distinguished. Heterotrophic and autotrophic denitrification could be assigned as preferred strategy to the mixotrophic incubation for *Acidovorax delafieldii* strain 16 and *Hydrogenophaga taeniospiralis* strain 2K1, respectively.

The demonstrated method offered the possibility to investigate the electron donor and carbon source preference of other facultative autotrophs in more detail using CERS headspace gas analysis in combination with stoichiometric calculations. Improved knowledge about metabolic activities in bacteria, kinetics and preferential substrate use can lead to a better understanding of dynamics of denitrification performed by natural microbial communities, allowing a more detailed understanding and better predictions of N cycling processes under different environmental conditions. This can help in assessing which substrates are likely to be used in natural environments and whether purely autotrophic, heterotrophic or rather mixotrophic processes will dominate. An example is the oligotrophic groundwater in Hainich National Park, which is poor in organic carbon and would call for autotrophic denitrification [22, 40]. But a mixotrophic lifestyle, where denitrification rates are enhanced by small amounts of organic carbon available to the bacteria, can also be imagined when the results of the experiments with *Hydrogenophaga taeniospiralis* strain 2K1 shown here are considered. These results do not apply to all bacterial strains, as can be seen from the comparison of denitrification performed by *Acidovorax delafieldii* and *Hydrogenophaga taeniospiralis*, and substrate preferences can differ greatly. This demonstrates the great versatility of denitrifying bacteria and requires a closer investigation of mixotrophic denitrification and substrate preference in denitrification. Cavity-enhanced Raman spectroscopy shows great prospects for the investigation of electron donor and carbon source preferences in denitrification.

## Supplementary information

ESM 1 (PDF 253 kb)
