# Supplementary material for: Activity and electron donor preference of two denitrifying bacterial strains identified by Raman gas spectroscopy
Source: Anal Bioanal Chem. 2021 Jul 23;414(1):601–11. doi: 10.1007/s00216-021-03541-y (PMC8748363; doi:10.1007/s00216-021-03541-y)
Supplement: Supplementary file 1 — (PDF 253 kb) [file 216_2021_3541_MOESM1_ESM.pdf]

Supplementary Information:
Activity and electron donor preference by two denitrifying bacterial strains identified by Raman gas spectroscopy

Annika Blohm[1], Swatantar Kumar[2], Andreas Knebl[1], Martina Herrmann[2,3], Kirsten Küsel[2,3], Jürgen Popp[1,4,5], and Torsten Frosch[1,4,5,6,*]

[1] Leibniz Institute of Photonic Technology, 07745 Jena, Germany
[2] Institute of Biodiversity, Friedrich Schiller University Jena, 07743 Jena, Germany
[3] German Centre for Integrative Biodiversity Research (iDiv) Halle-Jena-Leipzig, 04103 Leipzig, Germany
[4] Institute of Physical Chemistry, Friedrich Schiller University Jena, 07743 Jena, Germany
[5] Abbe Centre of Photonics, Friedrich Schiller University, 07743 Jena, Germany
[6] Biophotonics and Biomedical Engineering Group, Technical University Darmstadt, Merckstraße 25, 64283 Darmstadt, Germany

* torsten.frosch@tu-darmstadt.de, Orchid: 0000-0003-3358-8878

Figure S 1

An exemplary multi-gas spectrum recorded in the course of the experiment.

Figure S 2

Concentration changes of mannitol over time for heterotrophic denitrification by *Hydrogenophaga taeniospiralis.*

Methods

Measurement Procedures for mannitol

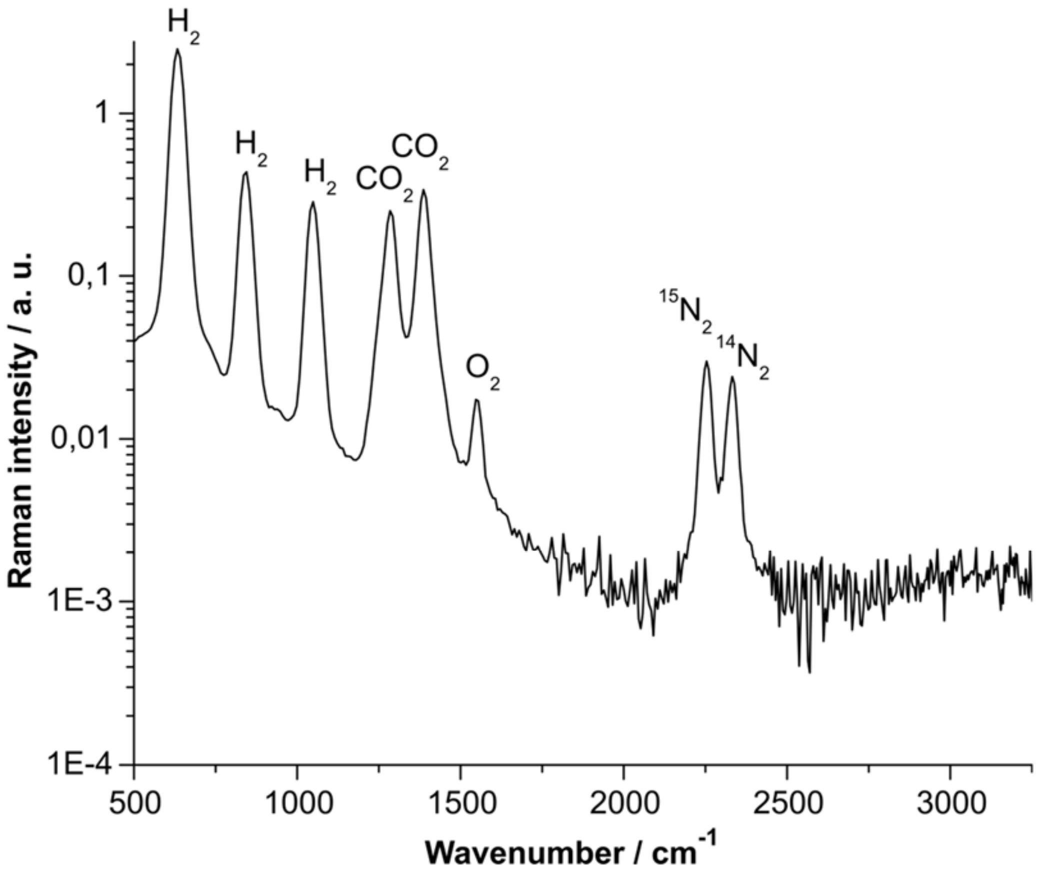

Figure S1: An exemplary multi-gas spectrum recorded in the course of the experiment. Rotational bands of H_2 ($S_0(1)$-$S_0(3)$) and vibrational bands of CO_2 (v_- and v_+), O_2, $^{14}N_2$ and $^{15}N_2$ (all v_0) can be clearly differentiated. Using linear combinations of calibration spectra of $^{12}CO_2$ and $^{13}CO_2$, the concentrations of both isotopologues can be gained from the Raman spectra.

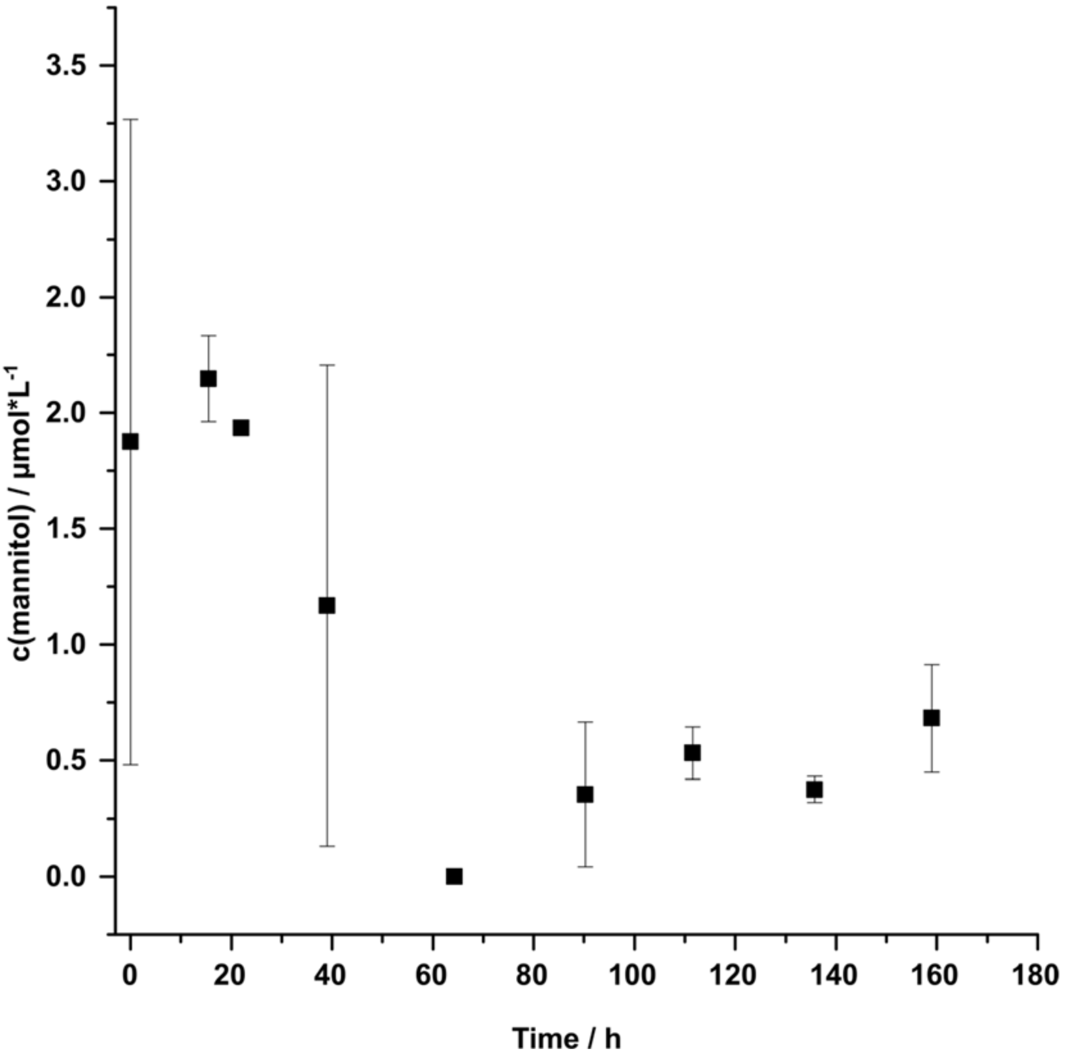

Figure S2: Concentration changes of mannitol over time for heterotrophic denitrification by *Hydrogenophaga taeniospiralis.* Depicted values are averaged measurements from three replicate culture flasks with standard deviation.

Methods

Measurement Procedure for Mannitol

Sample Preparation

10 µL of the respective culture broth were placed in a vial with insert and spiked with internal standard (40 µL of a 80 µM ribitol dissolved in water) and dried in an exicator over night at reduced pressure of 50 mbar followed by one hour at 5 mbar. The samples were dissolved in pyridine (20 µL) and treated with *N*,*O*-*bis*(trimethylsilyl)trifluoroacetamide BSTFA (20 µL) vortexed for 5 sec and kept at 60 °C for one hour. GC/MS analyses were measured subsequently in the order: blanks, calibration (increasing concentration), blanks, samples, blanks.

GC/MS measurement

Gas-chromatographic separations were executed on a THERMO Trace 1310 equipped with TriPlus RSH auto sampler and coupled with a THERMO TSQ 8000 electron impact (EI) triple quadrupole mass spectrometer. We used an Agilent DB-5ms+DG Columns column with the following dimensions: length 30+10 m; 0.25 mm inner diameter and 0.25 µm film. The column was operated with helium carrier gas using a S/SL injector operating with a column flow 1.2 mL min^{-1} and a split less injection for 1.5 min at a temperature of 300 °C. Them the split flow was set to a flow of 50 mL min^{-1}. The syringe was cleaned twice with 5 µL *n*-heptane each pre injection and rinsed with 1 µL sample before the injection was done. After injection, the syringe was washed five times with ethyl acetate and five times with *n*-heptane (5 µL each). The GC oven program starts at 80 °C for 2 min and the temperature was increased to 120 °C at 20 °C min^{-1}, and held for 1 min. Then the temperature was increased to 320 °C at 10 °C min^{-1}, and held for 1 min. The mass spectrometer started measuring after 10 min monitored the mass range between 50 and 650 *m*/*z*. The MS transfer line was set to 300 °C as well as the ion source temperature.

Data analysis was performed using Xcalibur Quan Browser 3.0.63. The specific ion at 217 *m*/*z* was used for integration. For mannitol the retention time range 16.88 min was used and for ribitol 14.66 min. Peak detection was performed in 30 sec windows. The ICIS peak detection algorithm was set to smoothing points: 1; baseline window: 40; Area noise factor: 5; peak noise factor 10; and maximum peak height (S/N): 3. For quantification a calibration curve was acquired with 100, 90, 80, 70, 60, 50, 40, 30, 20, 10, 5, 1% of the maximum expected mannitol concentrations. All data points have been recorded in triplicates, value 100% and 1% in quintuplicates.
